# Focus on endocrine surgery

**DOI:** 10.1186/s12893-019-0496-6

**Published:** 2019-04-24

**Authors:** Giovanni Conzo

**Affiliations:** University of Campania “Luigi Vanvitelli”, Via Sergio Pansini 5, 80131 Naples, Italy

It is a great honor for me to have the opportunity to introduce this special supplement to BMC Surgery, to celebrate the foundation in 2017, of a new prestigious Italian Endocrine Surgery Society: SIUEC (Società Italiana Unitaria di EndocrinoChirurgia) deriving from two famous Societies: Società Italiana di Endocrinochirurgia (SIEC) and Club delle Unità di EndocrinoChirurgia (Club UEC).

This monograph is meant to provide an overview of current knowledge and future developments of Endocrine Surgery. In the last decade, in this surgical field, novel and classic research topics have been investigated through the application of innovative technologies.

The development of less invasive surgical interventions, such as the trans-axillary and transoral thyroidectomy, the diffusion of new guidelines for the management of thyroid cancer and the identification of genetic mutations leading to prophylactic surgery are the main interesting innovations.

Neuroendocrine pancreatic tumors, whose incidence has been sharply increasing in the last few years, represent another important area of great interest deserving the investment of human and financial resources.

Italian contribution to Endocrine Surgery has deep historical roots. Articles included in this special supplement issue have been written by true expert Italian colleagues, with a deep knowledge in this field, confirming a high international reputation; readers will certainly find these studies as a useful tool for cultural update. The “Focus” presents the most recent findings about surgical treatment of Thyroid, Parathyroid, Adrenal and Pancreatic glands main pathologies.

Therefore I wish to thank the President and the Board of Italian Unitary Society of Endocrine Surgery for the honor of editing this “Focus on” and sincerely commend the papers’ authors for their strong commitment, and their precise analysis of the discussed pathologies. Their work will be an inspiration for future research.

Special thanks to Dr. Hayley Henderson, Editor of BMC Surgery, for allowing the publication of this special Supplement issue and to Miss Emma Robinson for the editorial assistance.

In conclusion, I need to express my sincere and affectionate gratitude to the co-Editors, Dr. Claudio Gambardella and Dr. Renato Patrone, and to the reviewers. The publication of this issue wouldn’t have been possible without their relentless efforts and support.

